# Structure improvement and parameter optimization of micro flow control valve

**DOI:** 10.1038/s41598-023-30955-8

**Published:** 2023-04-26

**Authors:** Guodong Qu, Jianying Li, Chen Peng, Qi Guo

**Affiliations:** 1 School of Mechanical and Power Engineering, Harbin University of Science and Technology, Harbin, 150080 China; 2 Key Laboratory of Advanced Manufacturing and Intelligent Technology, Ministry of Education, Harbin, 150080 China

**Keywords:** Electrical and electronic engineering, Mechanical engineering, Engineering

## Abstract

Aiming at the sticking phenomenon between the valve core and the valve sleeve when the valve core moves, and to solve the problem that the torque required to drive the valve core to rotate is large, the fluid–solid coupling simulation analysis of the valve core is carried out in this study, and then the valve core structure of the valve core is improved and its parameters are optimized based on the bird colony algorithm. The combination structure of the valve sleeve and valve core is studied, and the fluid–solid coupling model is established by Ansys WorkBench, and the static structure simulation analysis of valve sleeve and valve core before and after structural improvement and parameter optimization is performed. The mathematical models of triangular buffer tank, U-shaped buffer tank and combined buffer tank are established, and the structural parameters of the combined buffer tank are optimized by bird swarm optimization. The results demonstrate the triangular buffer tank has good depressurization effect but great impact, the pressure of the U-shaped buffer tank is stable and gentle but the depressurization effect is not ideal, while the combined buffer tank has obvious depressurization effect and good stability. At the same time, the optimal structural parameters of the combined buffer tank are cut-in angle of 72, plane angle of 60 and depth of 1.65 mm. The excellent structure and parameters of the combined buffer groove are obtained, so that the pressure buffer of the regulating valve at the key position of the valve port achieves the best effect, and an effective solution is provided for solving the sticking problem of the valve core of the regulating valve when working.

## Introduction

At present, the micro small hydraulic control valve is used more and more widely in Chin. Researchers and manufacturers at home and abroad have done a great deal of explorations on its theory, structure and parameters^1,2^. The regulator controls the fluid and drives the load by changing the opening of the valve port. Its regulating ability is of great significance to the safe and efficient operation of the hydraulic system^3^. Spool and valve sleeve this is extremely important to the structure of the working pair and the parameters of the control valve working ability. Luo Yuxuan et al.^4^ analyzed and summarized the causes of the stock spool in valve engineering applications. The reasons for the spool stuck are divided into: mechanical reasons caused by machining precision or assembly error; Hydraulic reasons caused by the unbalanced moment of fluid action on the spool; Thermal causes caused by viscous heating of fluid under high pressure conditions, and pollution causes caused by particle retention in valve fit clearance. Aiming at the stuck phenomenon of 2D electro-hydraulic proportional directional valve spool, Liu Guowen et al.^5^ systematically analyzed the radial clamping force of 2D spool with or without eccentricity. MATLAB software is used to calculate the relationship between 2D spool radial clamping force, eccentricity and angle between high and low pressure holes. Depending on the characteristics of 2D valve, the improvement measures of 2D electro-hydraulic proportional directional valve spool are put forward. The flow field on the valve core surface is simulated by CFD using Fluent software. The velocity vector and pressure distribution before and after the improvement is compared, and the correctness of the improved measures is verified. Pei Xiang et al.^6^ compared various clamping phenomena of rotary valve and slide valve spool. The radial unbalance force of the rotary valve is analyzed theoretically some concrete measures to reduce the clamping phenomenon of rotary valve is put forward. To provide some help for the design and application of a rotary valve in the future. Sun Zegang et al.^7,8^. Studied the influence of U-shaped and V-shaped throttling groove structures on the cavitation performance of the valve. By improving the throttling groove structure optimized by genetic algorithm, the anti-cavitation performance of the valve can be obviously improved. Li Weijia et al.^9^ studied the valve opening-flow characteristics of the slide valve with a single U-shaped, oblique U-shaped and V-shaped base throttling grooves under the condition of constant pressure difference, Using particle swarm optimization algorithm, the optimal size of the throttle slot is obtained, which meets the requirements of valve opening-flow characteristics under the condition of constant pressure difference. Cao Jia Hao et al.^10^ designed a new type of limiting structure with buffer groove, which weakened the structural rigidity and improved the damping dissipation of impact energy. Through ANSYS software, the parameter iterative optimization analysis of the original limit buffer structure is carried out to find a reasonable structure parameter combination. Then, the transient dynamic performance of the structure based on the time domain method is considered by LS-DYNA software, and the buffering effect of the traditional and new type limiting structures is compared. Wu Weidong et al.^11^ aiming at the problems of small flow adjustment range and slow response of the U-shaped throttling groove of a certain type of load sensitive valve, a Ω-shaped throttling groove was designed by analyzing the functional relationship between valve opening and flow area. Particle swarm optimization (PSO) algorithm is used to optimize its organizational parameters with flow gain as the goal. Zhang Zhandong et al.^12^ put forward a calculation method of adding a K-shaped throttle slot at the shoulder of radial flow hole of the main spool of the reversing valve, and obtaining the equivalent flow area of the K-shaped throttle slot, aiming at the situation that the valve port of the reversing valve of coal mine hydraulic support has a large flow area gradient, which can only realize the on–off control function, and the pressure build-up is sudden, which is easy to induce the pressure impact of the support oil supply system. The purpose of actively regulate and controlling that flow area gradient of the valve port can be realized. Zhang Liqiang et al.^13^ aimed at the problem of valve port pressure impact caused by excessive internal flow of a slide valve. After analysis the influence of throttle groove structure parameters on its flow characteristics. The genetic optimization algorithm is used to obtain the Parato solution set of the U-shaped throttling groove which meets the fast response characteristics of flow and the pressure impact performance. The optimization results are verified by selecting specific throttle groove structure parameters. Li Ping^14^ put forward an improved scheme of a new type of throttling tank (U–V combined tank), particle swarm optimization algorithm is used to optimize the structure of throttle groove, the optimal structural parameters are obtained. Under the same valve opening, the flow area of the new throttle groove is larger than that of the original value (U-shaped groove). When the multi-channel valve reaches the rated flow rate, the opening of the new valve decreases and the flow adjustment range increases. Yi Sheng et al.^15^ carried out simulation research on the opening-flow characteristics of six kinds of throttling slots: single U-shaped, oblique U-shaped, V-shaped, 2U-shaped, 3U-shaped and U + V-shaped; Using GUI module in MABLAT, the optimization design software of throttle slot based on particle swarm optimization is developed. Fang Guihua et al.^16^ studied the influence of different parameters on the steady-state hydrodynamic force of the U-shaped throttling groove, and thought that the height of the U-shaped throttling groove had greater impact than the width.

Through analysis, it can be known that under normal working conditions, the pressure impact in the regulating valve, especially at the key position of the valve port, is easy to occur, which leads to the deformation of the valve core, and then leads to the clamping phenomenon between the valve core and the valve sleeve when it moves. To solve this problem, In the concrete analysis, the structural model of the regulating valve is simplified first, Then a mathematical model is established to calculate and study it, The calculation and analysis results of triangular buffer tank and U-shaped buffer tank is obtained, Then, the combined buffer tank is proposed, and the parameters of the combined buffer tank are optimized by the bird colony algorithm. Thereby improving the original valve core structure. Finally, through the comparative analysis of the fluid–solid coupling simulation before and after the valve port structure optimization of the regulating valve. It is concluded that the combined buffer groove can reduce the compression deformation of the valve core.

## Methods

### Structure principle of regulating valve

**Figure 1:**
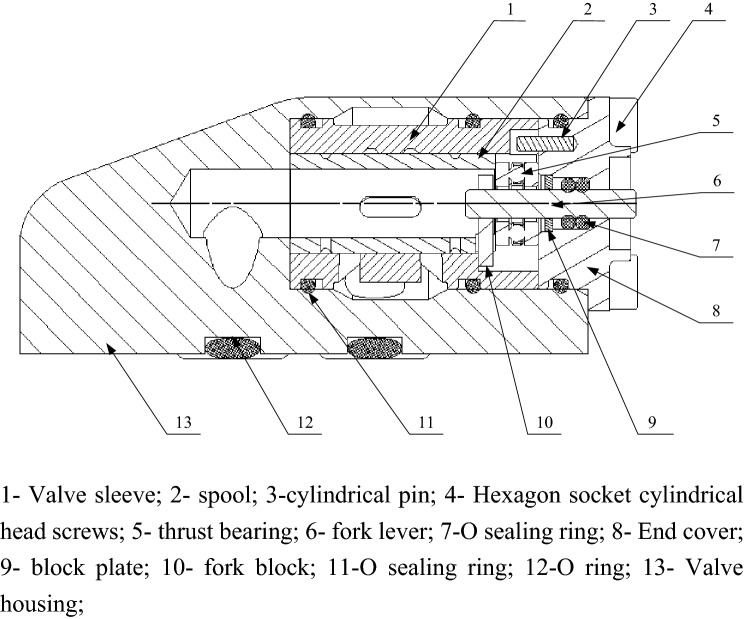
Two-dimensional assembly drawing of micro flow regulating valve.

This paper describes a micro-control valve with adjustable flow rate. Its working mechanism belongs to the category of hydraulic rotary valve, and its structure is shown in Fig. 1. It is mainly composed of shift lever, shift block, thrust bearing, valve sleeve and valve core. Micro-flow regulating valve controls the flow rate by driving the shift lever to rotate through the motor, and then adjusting the opening size by rotating the valve core through the shift block.

**Figure 2:**
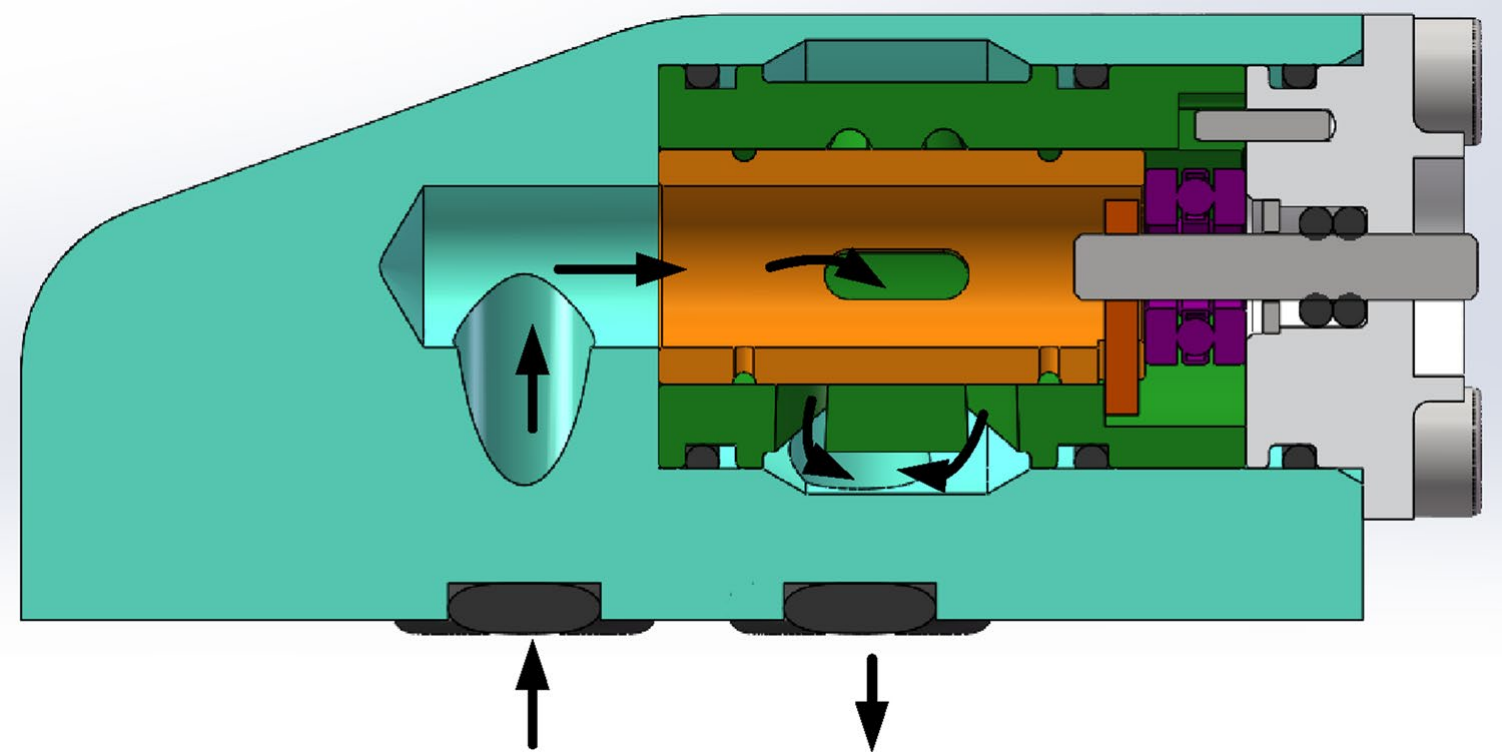
Oil flow diagram of micro flow control valve.

Micro-flow regulating valve controls the outlet flow by adjusting the opening size through the rotation of motor valve core. As showed in Fig. 2, it can be seen from the arrows in the three-dimensional diagram that the oil enters the valve from the inlet, flows through the flow passage in the valve housing, and then flows into the valve core. When the valve core rotates, the valve port of the valve core coincides with the valve port of the valve sleeve, so that the oil in the valve core flows out of the valve sleeve from the coincidence position, and then flows through the valve housing flow passage and out of the oil outlet. The overall fluid flow direction is consistent with the arrow in the figure.

**Figure 3:**
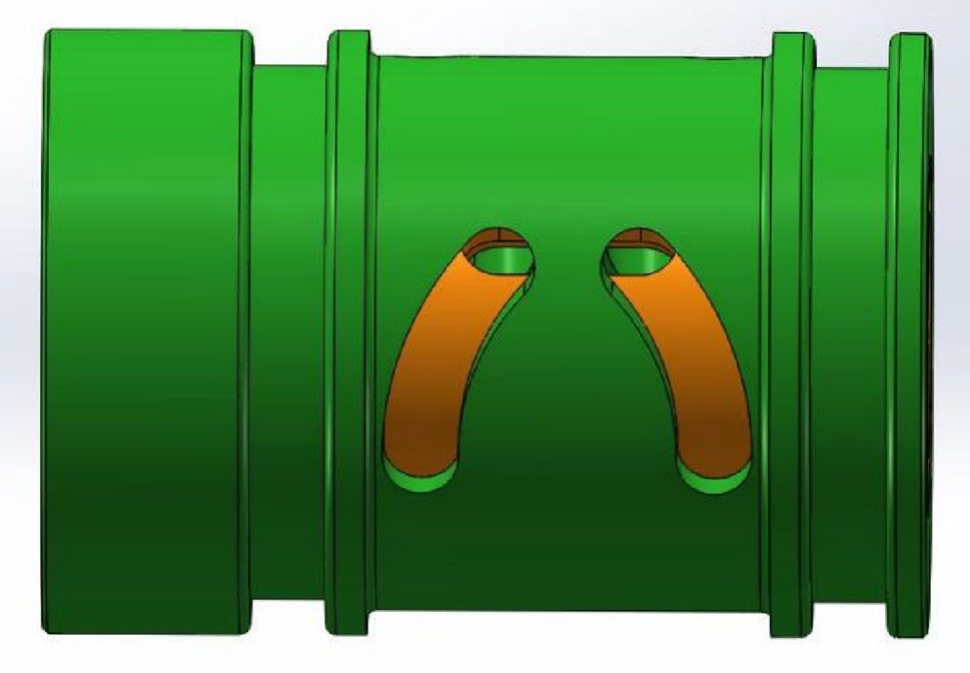
Combination structure of valve sleeve and valve core.

As showed in Fig. 3, the three-dimensional model of the combination of valve sleeve and valve core is established through the above parts drawings. The working principle of the valve sleeve is as follows: a splayed waist-shaped valve port is arranged on the same annular surface of the valve sleeve at intervals of 120 degrees, and an annular groove valve port is arranged on the annular surface of the valve core at intervals of 120°, and every two adjacent valve ports on the valve sleeve correspond to an annular valve port on the valve core. As the spool rotates, the coincidence degree between them will change.

**Figure 4:**
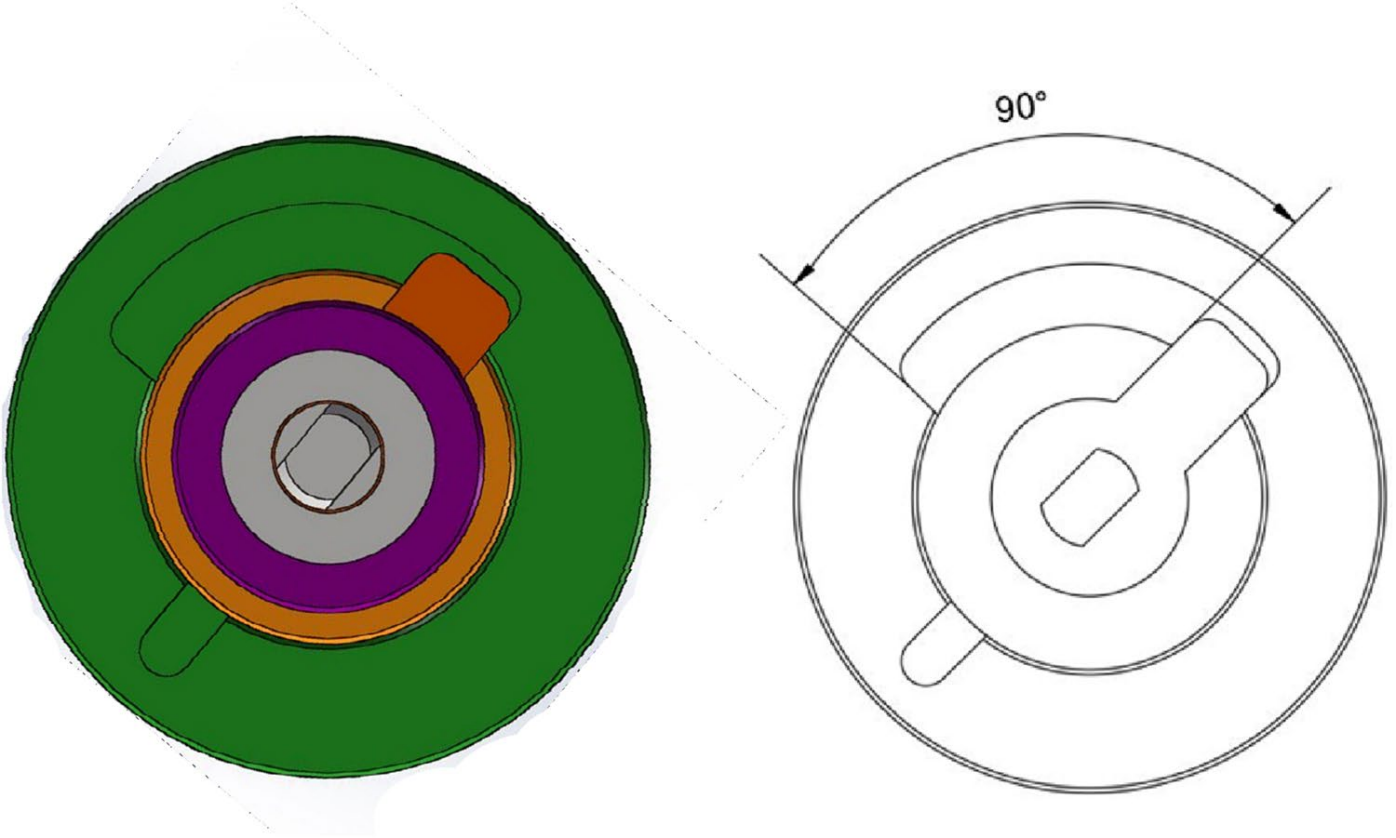
Working section of rotary valve.

The servo motor drives the shift lever to rotate, so that the shift fork block reciprocates in the arc groove at the bottom of the valve sleeve. The angle of the arc groove at the bottom of the valve sleeve is 90, so the maximum rotation angle of the valve core is 90, as showed in Fig. 4. When the working valve core reciprocates circumferentially in the arc groove, the opening coincidence degree of the valve sleeve and the valve core will change, and the oil quantity can be adjusted.

### Improvement of valve port structure of regulating valve

Taking the sealed cavity formed by the valve core and the valve sleeve as the research object, the pressure change in the valve port cavity can be calculated by the following formula:

1 $$dp = - \beta_{e} \frac{dV}{V}$$

where *dp* is the oil pressure differential of the valve port cavity of the regulating valve; *dV* is the oil volume differential of the valve port cavity of the regulating valve; *βe* is the elastic modulus of oil (MPa); *V* is the initial volume (m^3^) of oil in the orifice cavity of the regulating valve.

Assuming that the initial volume of the valve port volume of the regulating valve is V, the pressure formula is derived according to Eq. (1), and the expression is:

2 $$\frac{dp}{{dt}} = \omega \frac{dp}{{d\theta }} = - \beta_{e} \frac{{\frac{dV}{{dt}}}}{V}$$

where: *ω* is the speed of the regulating valve (°/s); *θ* is the rotor angle (°) of the regulating valve.

The volume of oil in the valve port volume can be represented by $dV/dt$, the pressure gradient formula of the oil in the valve port cavity of the regulating valve is as follows.

3 $$\frac{dp}{{d\theta }} = - \frac{{\beta_{e} }}{\omega }\frac{{\frac{dv}{{dt}}}}{V}$$

### Mathematical model of valve port cavity with triangular buffer grooves structure

**Figure 5:**
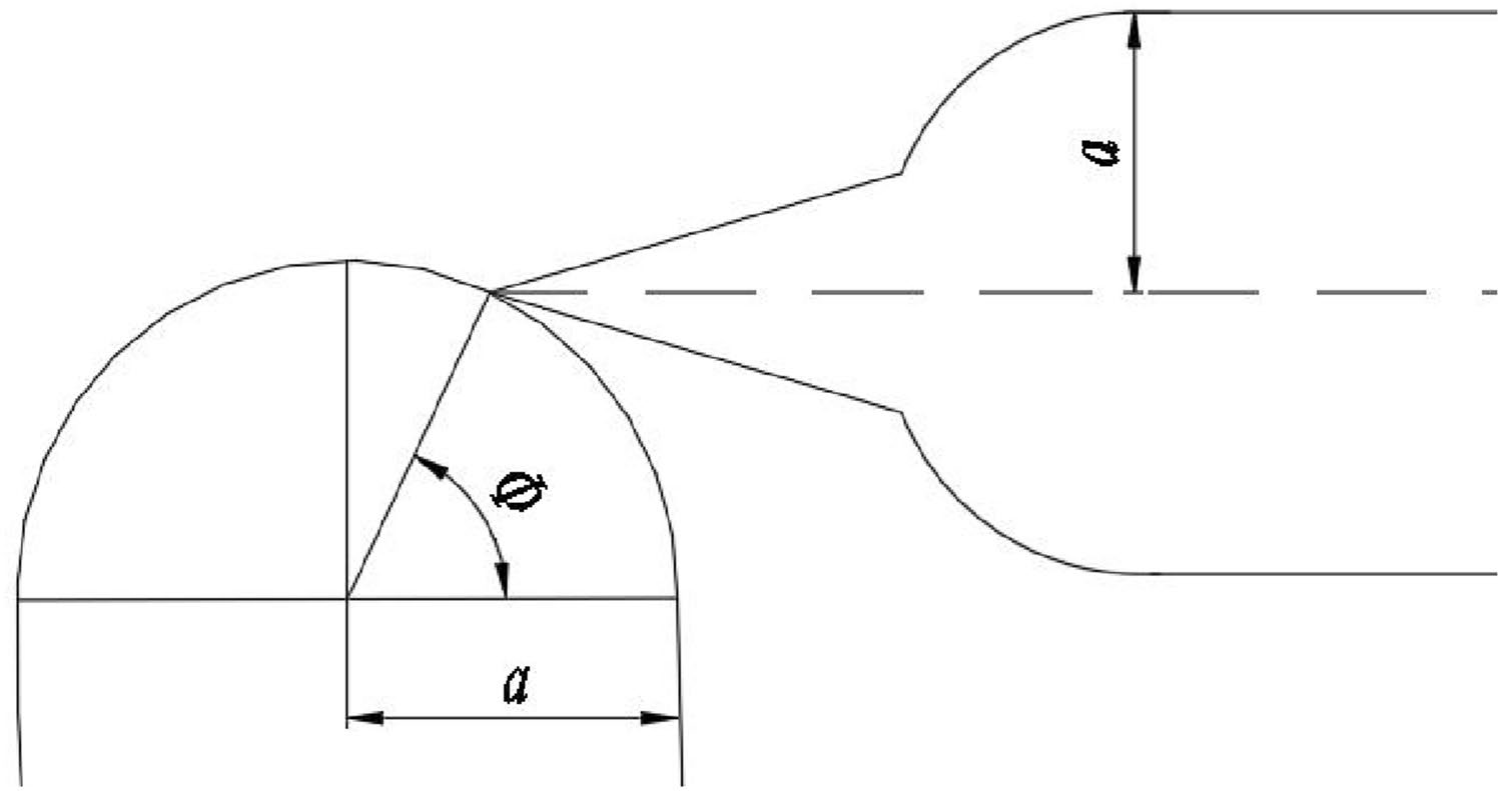
Schematic diagram of triangle buffer groove tangent to valve sleeve waist groove.

The three-dimensional structure of triangular buffer groove can be regarded as a triangular vertebral body, and its plane principle is the relative movement of triangle and circle. Its size is controlled by two parameters: cutting angle Ф and plane angle α, as showed in Fig. 5.

**Figure 6:**
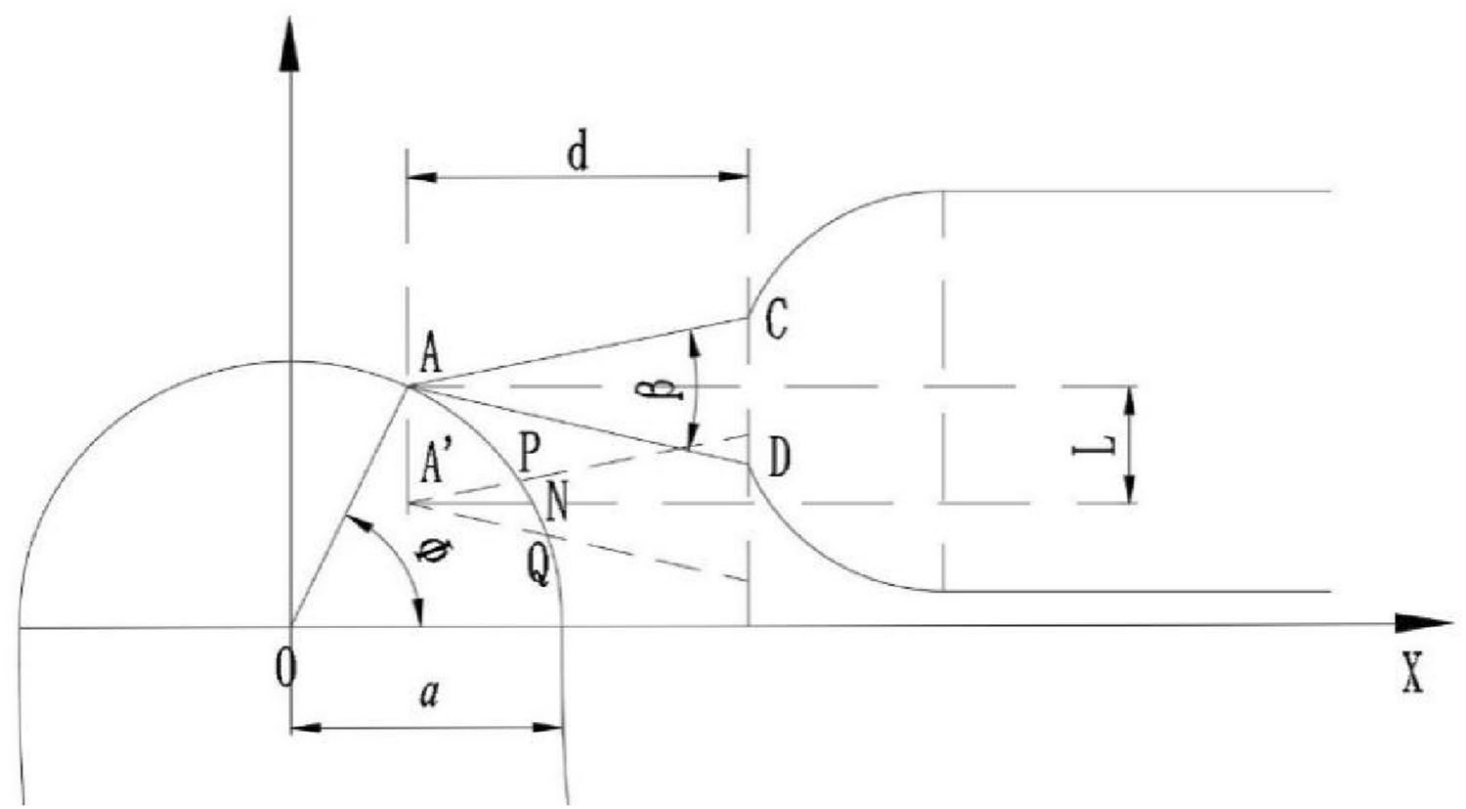
Coordinate diagram of triangular buffer tank model establishment.

The triangular buffer groove is set at the end of the valve port of the U-shaped groove of the valve core. Firstly, the rotary motion of the valve core is expanded into a plane motion, which is equivalent to the rotary angle of the valve core. After expansion, it can be seen as the downward displacement L of the U-shaped groove of the valve core. Then, the relative motion of the U-shaped groove of the valve core and the waist-shaped groove of the valve sleeve is expanded into a plane motion, which is simplified into a mathematical model. Then, the geometric model is established for analysis and solution, as showed in the Fig. 6.

If the cross section of a triangular pyramid is always an obtuse triangle, the relationship between the depth angle and the plane angle is as follows, and the relationship can be expressed as:

4 $$L = r\theta$$

5 $$y = \sqrt {a^{2} - x^{2} }$$

6 $$y_{1} = a\sin \upvarphi - r\theta + \tan \frac{\upbeta }{2}(x - a\cos \upvarphi )$$

7 $$y_{2} = a\sin \upvarphi - r\theta + \tan \frac{\upbeta }{2}(x - a\cos \upvarphi )$$

Simultaneous solution Eqs. (5)–(7),can be solved separately P(x_P_, y_P_), Q(x_Q_, y_Q_). Substitute two points *P* and *Q*. The *PQ* linear equation can be obtained as follows:

8 $$y - y_{p} = \frac{{y_{p} - y_{Q} }}{{x_{p} - x_{Q} }}(x - x_{p} )$$

**Figure 7:**
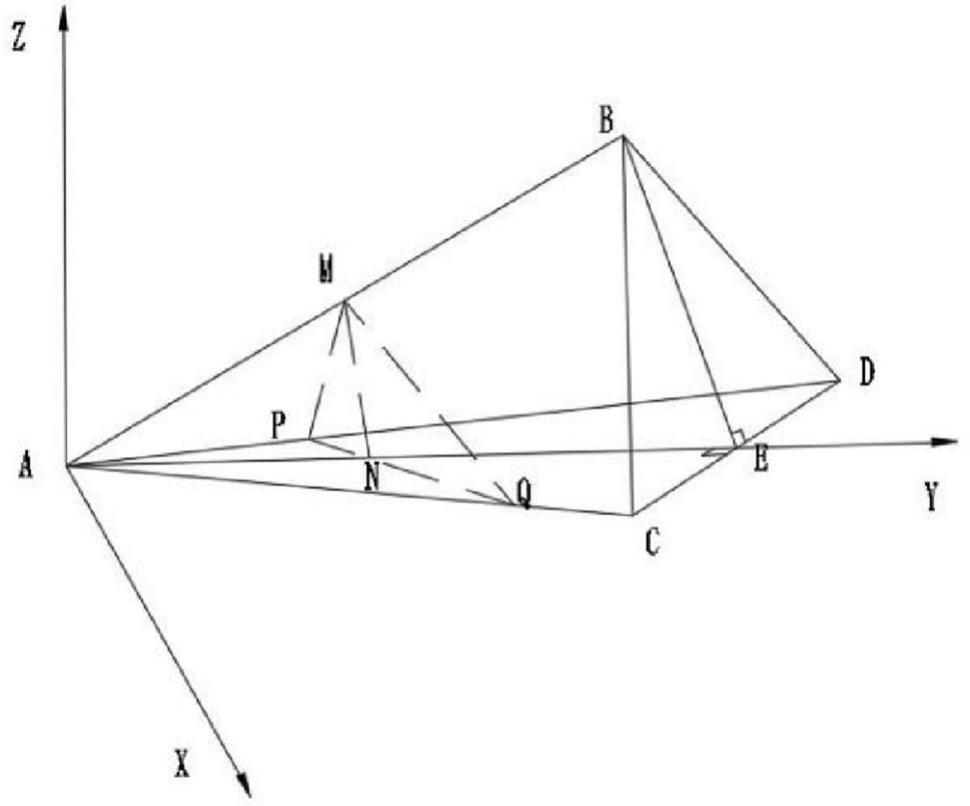
Spatial coordinate system of mathematical model of triangular buffer tank.

As showed in Fig. 7, the triangular buffer groove on the valve core simplifies the mathematical model of the triangular buffer groove. In this state, the space rectangular coordinate system is established, in which the plane *∆ADC* coincides with the working plane of the U-shaped valve port of the valve core, In the process of turning the waist groove of the valve sleeve into the triangular buffer groove, Where any cross section *∆PQM* is an obtuse triangle, the area of *∆PQM* is defined as the flow area of the triangular groove.

For triangle whose cross section is kept obtuse, that relationship between depth angle and plane angle is as follows:

9 $$d = a(1 - \cos \varphi )$$

10 $$BE = d \cdot \tan \alpha = \sqrt 3 CE$$

11 $$CE = d \cdot \tan \frac{\upbeta }{2}$$

12 $$\tan \upalpha = \sqrt 3 \tan \frac{\upbeta }{2}$$

Then, a space rectangular coordinate system is established with point of A(a cos φ, a sin φ − rθ) as the coordinate origin, and the over-current area *∆PQM* is solved.

The equation can be obtained by substituting the point of N(xN, a sin φ − rθ) into the linear equation *PQ*. Suppose the point of M(x_N_, y_N_, z), as can be seen from Fig. 7. Finally, by combining the above equation, the expression of triangular buffer slot can be obtained as follows Eq. (15).

13 $$x_{N} = \frac{{a\sin \upvarphi - r\theta - y_{p} }}{{y_{p} - y_{Q} }}(x_{p} - x_{Q} ) + x_{p}$$

14 $$z = (x_{N} - a\cos \upvarphi )\tan \upalpha$$

15 $$A = \frac{1}{2}\left| {\overrightarrow {PQ} \times \overrightarrow {PM} } \right|$$

As the regulating valve is a micro-hydraulic component, the volume change of the triangular buffer groove flowing into or out of the valve port cavity of the regulating valve can be obtained according to the flow formula of the throttle orifice:

16 $$\frac{{dV_{{}} }}{dt} = C_{q} A\sqrt {\frac{2\Delta p}{\rho }}$$

where: *Cq* is the flow coefficient; Is the inlet and outlet pressure difference of regulating valve (MPa); *A* is the throttling area of the valve port cavity; *ρ* is the density of hydraulic oil (kg/m^3^).

According to the above formula, When the volume of the triangular buffer groove flowing into or out of the valve port cavity changes into the operation of the regulating valve, The cavity of the valve port is surrounded by a valve sleeve and a valve core, and the volume of oil in the cavity is based on Eqs. (15) and (16). The pressure gradient equation of the sealing chamber of the valve port of the regulating valve under the triangular buffer groove structure can be obtained:

17 $$\frac{dp}{{dt}} = - \beta_{e} \frac{{C_{q} \sqrt {\frac{{2\Delta p}}{\rho }} }}{V}A$$

In the formula, only the over-current area is a variable value, and the rest are fixed values, which can be set:

18 $$K = - \beta_{e} \frac{{C_{q} \sqrt {\frac{{2\Delta p}}{\rho }} }}{V}$$

19 $$\frac{dp}{{dt}} = K \cdot \frac{1}{2}\left| {\overrightarrow {PQ} \times \overrightarrow {PM} } \right| = K \cdot A$$

**Figure 8:**
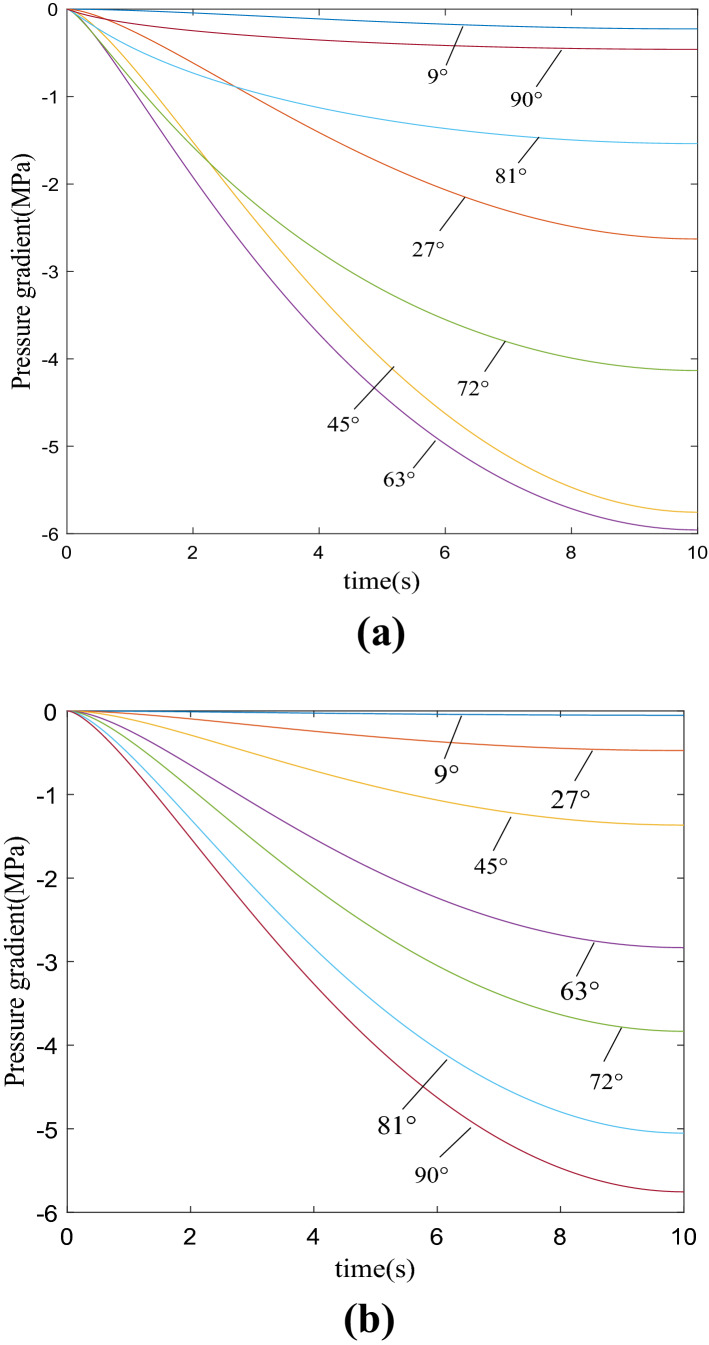
Curve of pressure gradient of triangular buffer tank with time.

According to the above theoretical analysis, numerical simulation is carried out with MATLAB, and the curves of pressure gradient with time under different cutting angles and different plane angles under triangular buffer groove structure can be obtained by calculation, as showed in Fig. 8. From the figure, the pressure gradient in the valve port cavity of the valve sleeve and valve core combination structure gradually increases with the rotation of the valve core. Figure 8a shows the curve of pressure gradient obtained by changing different cutting angles when the plane angle is set to a fixed value. In the cutting angle Ф = 63°, although the pressure gradient is large, the pressure impact is relatively large. When the pressure gradient changes greatly and the pressure impact is small, the optimal cutting angle should be selected Ф = 72°. Figure 8b shows the pressure gradient curve obtained by changing different plane angles when the cutting angle is set to a fixed value. When considering the need for a certain pressure gradient and minimizing the pressure impact, the plane angle should be selected *β* ϵ (45°, 81°). Therefore, considering the pressure gradient curve results comprehensively, it can be obtained that the triangular buffer groove has the best depressurization effect at the valve port cavity of the regulating valve at the cutting angle Ф = 72° and plane angle *β* ϵ  (45°, 81°).

### Mathematical model of valve port cavity with U-shaped buffer groove structure

As for the U-shaped buffer tank, it can be seen as consisting of a semi-cylindrical section and an equal cross-sectional area section, The size of its structure can be determined by width b and depth h, The U-shaped buffer groove is arranged at the U-shaped valve port of the valve core. When the U-shaped groove is opened in the semi-cylindrical section, the flow area increases rapidly, and when it transits to the section with equal cross-sectional area, the flow area remains unchanged.

**Figure 9:**
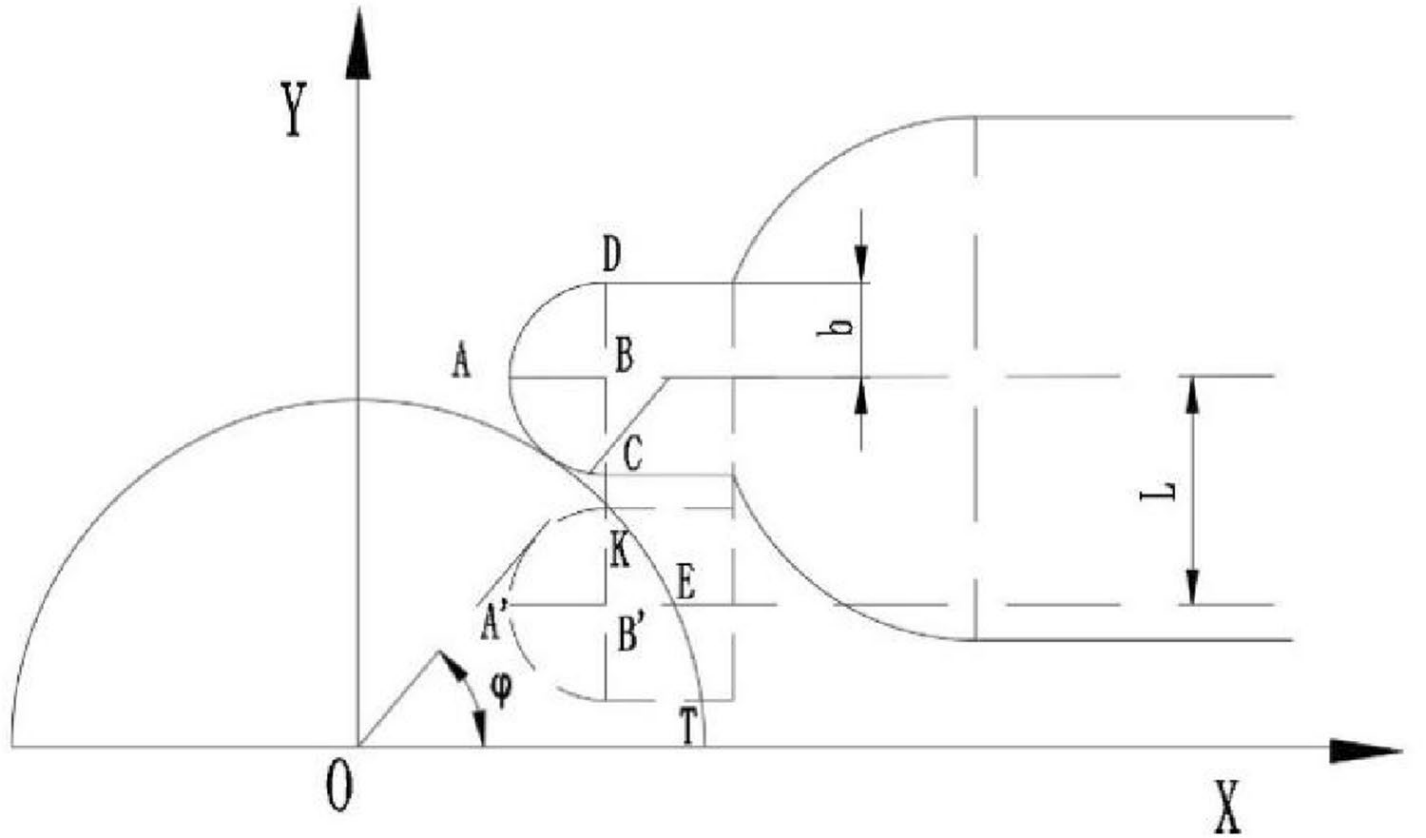
U-shaped buffer tank model establishment coordinate diagram.

As showed in Fig. 9, at this time, we define that when the U-shaped buffer groove just comes into contact with the waist-shaped groove, The angle between the tangent point and the center of the circle and the horizontal direction is that the tangent angle of the rotated semi-cylindrical segment is φ, the depth and width of the U-shaped buffer groove are b, and a rectangular coordinate system is established.

As showing in Fig. 9, the established coordinate system can be obtained:

20 $$a\cos \upvarphi + b\cos \upvarphi < a$$

21 $$b < \frac{a(1 - \cos \upvarphi )}{{\cos \upvarphi }}$$

These variables axis φ ∈ (0, π/2). It can be obtained through the calculation of the above formula:

22 $$b \in \left( {0,\frac{a(1 - \cos \varphi )}{{\cos \varphi }}} \right)$$

23 $$CE = BE - b$$

24 $$DE = BE + b$$

25 $$BE = (a + b)\sin \varphi - \sqrt {a^{2} - (a + b)^{2} \cos^{2} \varphi }$$

26 $$CE = (a + b)\sin \varphi - \sqrt {a^{2} - (a + b)^{2} \cos^{2} \varphi } - b$$

27 $$DE = (a + b)\sin \varphi - \sqrt {a^{2} - (a + b)^{2} \cos^{2} \varphi } + b$$

Establish the rectangular coordinate system of the simplified model. Let the equation of semicircle O and the equation of semicircle* O*_*B*_:

28 $$x^{2} + y^{2} = a^{2}$$

29 $$(x - x_{B} )^{2} + (y - y_{B} )^{2} = b^{2}$$

**Figure 10:**
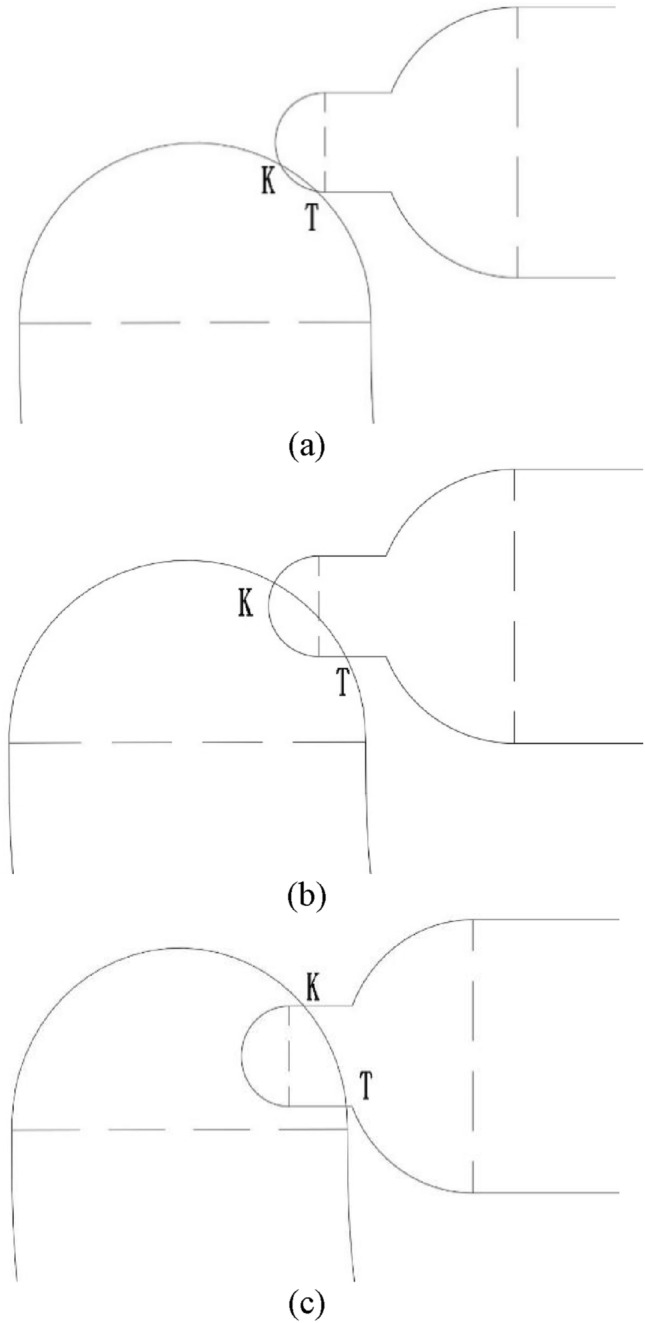
Theoretical analysis diagram of tangency between U-shaped buffer groove and waist groove of valve sleeve.

Among them, h is the groove depth of the U-shaped buffer groove. According to geometric analysis, the area *S* is mainly related to the length of *KT*, but it can be divided into three cases for the straight line *KT*, as showed in Fig. 10.

When *L* ∈ [0, CE], as showed in Fig. 10a, it can be seen that two semicircles intersect at two points *K* and *T*.

30 $$x_{B} = (a + b)\cos \varphi$$

When *L* ∈ [CE, DE], as showed in Fig. 10b, it can be seen that the point *K* intersect the circle, while the point *T* intersects the straight line.

31 $$x_{T} = \sqrt {a^{2} - y_{T}^{2} }$$

32 $$y_{T} = (a + b)\sin \varphi - r\theta - b$$

When *L* ∈ [DE, (a + b) sin φ], as shown in Fig. 10c, it can be seen that two points *K* and *T* intersect with a straight line.

33 $$x_{K} = \sqrt {a^{2} - y_{K}^{2} }$$

34 $$y_{K} = (a + b)\sin \varphi - r\theta + b$$

35 $$x_{T} = \sqrt {a^{2} - y_{T}^{2} }$$

36 $$y_{T} = (a + b)\sin \varphi - r\theta - b$$

37 $$y_{B} = (a + b)\sin \varphi - r\theta$$

Simultaneous solution of the above equation can lead to point *K*(x_K_, y_K_) and *T*(x_T_, y_T_); through simplifying model analysis, it can be concluded that the overflow area of the U-shaped buffer tank. The following formula is obtained:

38 $$\left| {\overrightarrow {KT} } \right| = \sqrt {(x_{K} - x_{T} )^{2} + (y_{K} - y_{T} )^{2} }$$

39 $$S = \left| {\overrightarrow {KT} } \right| \cdot h$$

According to the same principle, for the pressure gradient of the valve port cavity, only the flow area is a variable value, and the rest is a fixed value. When the volume of the valve port sealed cavity flowing into or out of the U-shaped buffer groove changes, the valve port cavity is composed of a valve sleeve and a valve core structure, and the oil volume in the cavity can be deduced according to formulas (17), (18) and (39), and the pressure gradient equation in the valve port cavity of the regulating valve with the U-shaped buffer groove structure can be obtained:

40 $$\frac{dp}{{dt}} = K \cdot \left| {\overrightarrow {KT} } \right| \cdot h = K \cdot S$$

According to the above analysis, the mathematical model is written by MATLAB, and the pressure gradient curve of U-shaped buffer groove with the same depth and different cutting angles and the pressure gradient curve of U-shaped buffer groove with the same cutting angle and different depths can be obtained after calculation.

**Figure 11:**
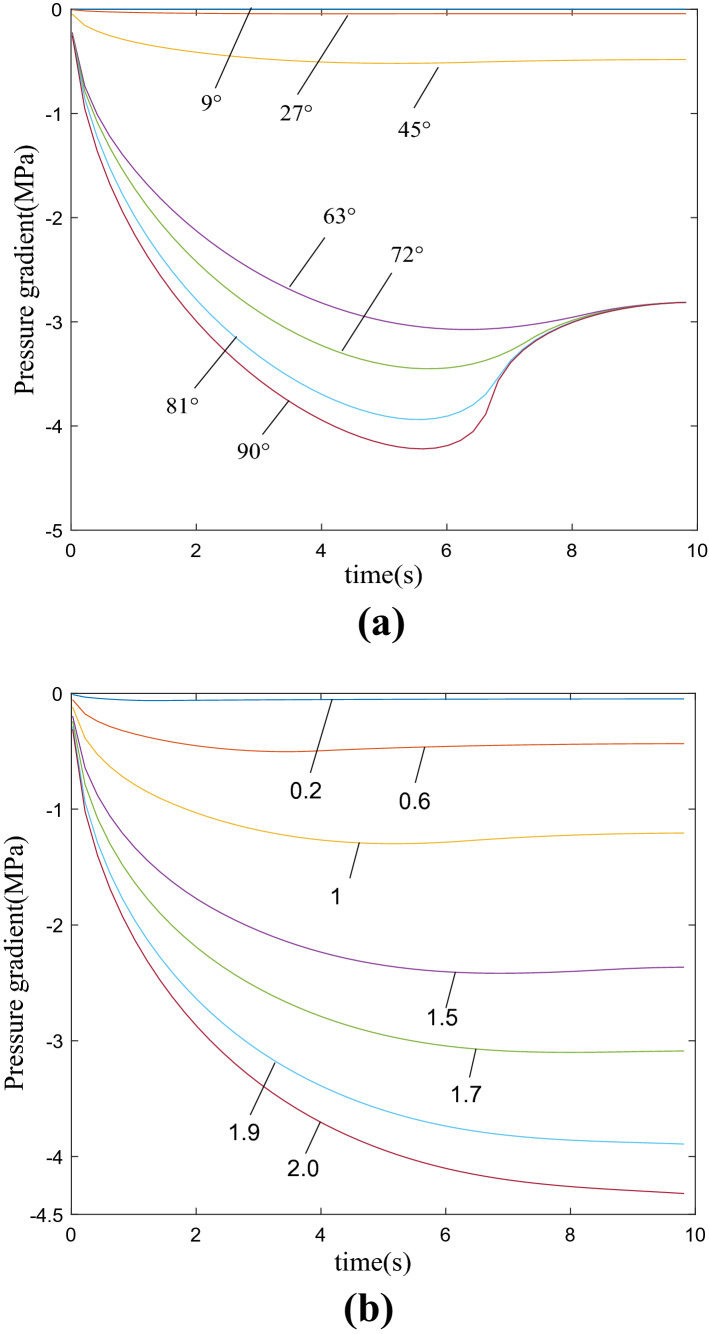
Curve of pressure gradient of u buffer tank with time.

As showed in Fig. 11a, it can be seen that the pressure gradient in the valve port cavity gradually increases with the rotation of the valve core. When the cutting angle ${\varphi }=72^\circ$, it is the best cut-in angle when the pressure gradient changes greatly and the pressure impact is small. In Fig. 11b, as the depth h gradually increases, the pressure gradient also gradually increases. When considering the need for a certain pressure gradient. Reduce the pressure impact as much as possible, the depth *h* ∈ (1.5–1.7) should be selected. Therefore, comprehensive consideration of the pressure gradient curve results shows that when the cutting angle ${\varphi }=72^\circ$ and the depth *h* ∈ (1.5–1.7), the U-shaped buffer groove has the best depressurization effect at the valve port of the regulating valve.

### Design of bird swarm optimization algorithm

Bird swarm optimization algorithm is a new biological heuristic optimization algorithm proposed by Xian-Bing Meng et al. ^17^. The algorithm is based on swarm intelligence evolved from the social behavior and social interaction of birds, and birds share information among groups through foraging behavior, alert behavior and flying behavior in their living habits^18,19^. Studying these social behaviors and interactions can effectively optimize the problem, and has the advantages of fast convergence and avoiding falling into local optimum^20,21^. Simplify the social behavior of birds into the following rules.

1. In the process of alerting and foraging behavior of birds, each bird can arbitrarily change its alert behavior and foraging behavior;
2. In the process of foraging, in order to get the best experience of finding food. Every bird can record and update individuals and groups.
3. Birds try to move to the center of the group while keeping alert. Affected by the competition among birds, birds with high reserves are more likely to approach the center of the flock than those with low reserves.
4. Birds randomly switch between production and search, and regularly fly to another location. The producer is the bird with the highest reserves, otherwise it grows up to be a requester. Other birds will randomly choose between the producer and the requester.
5. In the process of bird looking for food, birds are randomly divided into requesters and producers, and the requesters will follow the producers to find food.

Behaviors under each rule^22,23^ are stated: suppose there are n birds in the B-dimensional space. At the position at time T, individuals are searching for food in groups, that is, the position of the bird at time T can be expressed as *X*_*i*_(*i* ∈ 1, 2, … *N*).

Foraging behavior: when each bird searches for food through its own different experience, it can write a mathematical way according to rule 2:

41 $$\begin{aligned} x_{i,j}^{t + 1} & = x_{i,j}^{t} + \left( {p_{i,j} - x_{i,j}^{t} } \right) \times C \times {\text{rand}}(0,1) \\ & \quad + \left( {g_{j} - x_{i,j}^{t} } \right) \times S \times {\text{rand}}(0,1) \\ \end{aligned}$$

42 $$\begin{aligned} x_{i,j}^{t + 1} & = x_{i,j}^{t} + A1\left( {{\text{mean}}_{j} - x_{i,j}^{t} } \right) \times {\text{rand}}(0,1) \\ & \quad + A2\left( {p_{k,j} - x_{i,j}^{t} } \right) \times {\text{rand}}( - 1,1) \\ \end{aligned}$$

43 $$A1 = a_{1} \times \exp \left( { - \frac{{pFit_{i} }}{{{\text{ sumFit }} + \varepsilon }} \times N} \right)$$

44 $$A2 = a_{2} \times \exp \left( \begin{gathered} \left( {\frac{{pFit_{i} - pFit_{k} }}{{\left| {pFit_{k} - pFit_{i} + \varepsilon } \right|}}} \right) \hfill \\ \times \frac{{N \times pFit_{k} }}{{{\text{ sumFit }} + \varepsilon }} \hfill \\ \end{gathered} \right)$$

45 $$x_{i,j}^{t + 1} = x_{i,j}^{t} + rand(0,1) \times x_{i,j}^{t}$$

46 $$x_{i,j}^{t + 1} = x_{i,j}^{t} + (x_{k,j}^{t} - x_{i,j}^{t} )FL \times rand(0,1)$$

The pressure change in the valve port cavity during the rotation of the control valve is studied. According to the pressure gradient equation of the sealed cavity under the combined pressure groove structure, the pressure in the valve port cavity changes. Therefore, in order to obtain the parameters of the combined pressure tank with better pressure reduction effect in the process of pressure reduction, the pressure in the valve port cavity of the regulating valve is simulated and calculated by using the bird swarm optimization algorithm with different cut-in angles and depth dimensions.

**Figure 12:**
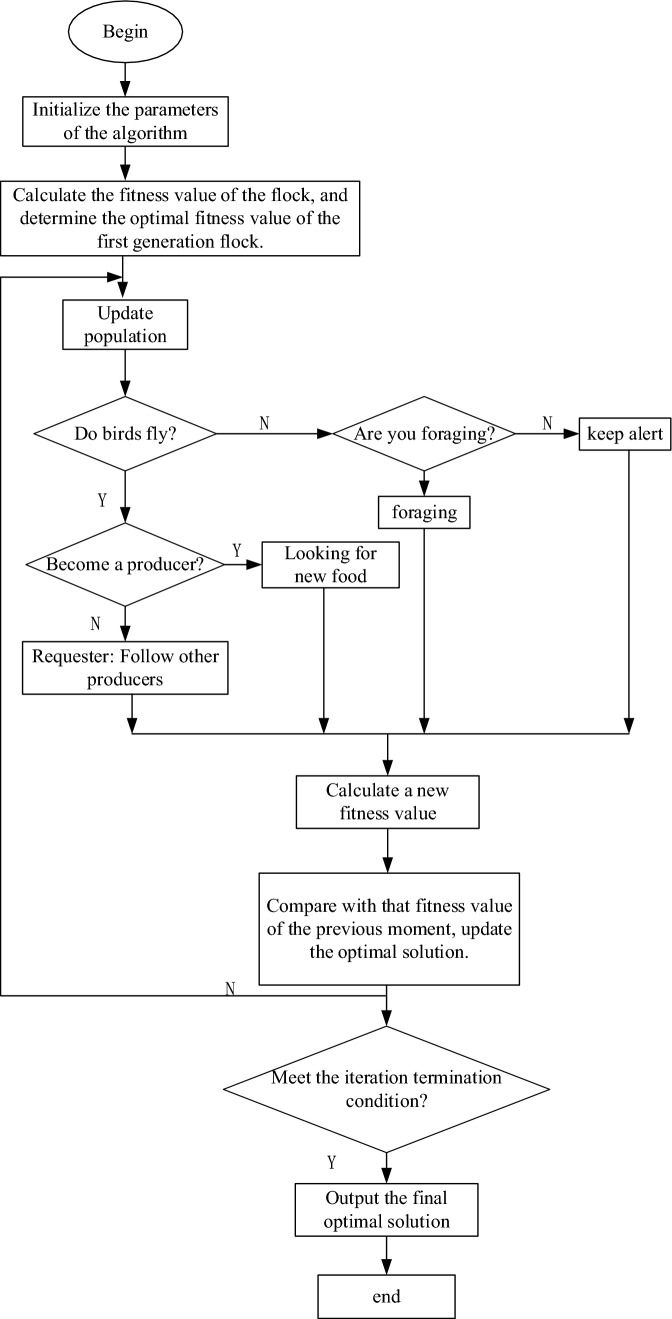
Flow chart of bird swarm optimization algorithm.

This paper aims at the pressure gradient model of the valve port volume of the regulating valve under the combined tank, and optimizes the parameters of the combined pressure tank according to the flow chart of the algorithm^24,25^ of the bird swarm optimization algorithm as showed in Fig. 12. The specific process is as following.

1. Initialize the basic parameters of the bird swarm optimization algorithm, such as the total number of birds, the particle dimension of birds, the maximum number of iterations, the flying frequency of birds, the foraging probability and other parameters, and generate the population under various constraints.
2. Set the pressure gradient equation of the regulating valve in the depressurization process as the objective function, According to the position ${x}_{i,j}^{t}$ of the flock, mark it as (*Ci*). According to the objective function, the fitness value *F(x)* corresponding to the position of each bird is calculated, and the initial optimal fitness value *F(x*_*1*_*)*_*min*_ and *bestlndex* are selected from the fitness values as the code corresponding to its optimal fitness value.
  47 $$F\left( x \right) = FitFun\left( {x_{1} } \right)_{\min }$$
  48 $$[{\text{fmin bestIdex}}] = {\text{F}}({\text{x}}_{1} )_{\min }$$
3. According to the update strategy corresponding to the biological behavior of the birds, update the birds *rand(0,1)* > *pi*, birds choose to perform foraging behavior in social behavior, and update according to the formula; if *rand(0,1)* > *pi*, according to the bird type Eq. (48) to carry out the alert behavior. Skip to step 4 after step 3 is completed.
4. Update the individual fitness value of the flock again, and compare it with the fitness value calculated at the previous moment, so as to update the optimal fitness value of the next generation flock *F(xi)*_*min*_. When the number of iterations *i* = *1* and *glbol_best* = *F(xi)*_*min*_; Compare the sizes of *glbol_best*_*i−1*_ and *F(xi)*_*min,*_*glbol_best*_*i*_ = *min{glbol_best*_*i−1*_*,F(xi)min}*.
5. Judging whether the iteration meets the iteration termination condition. *i* = *i* + *1* is executed, and the number of iterations *i* at this moment is compared with the maximum number of iterations t_max_. If *i* < *t*_*max*_ jump back to step 3; otherwise, the optimization is finished.

Generally, the iteration termination condition is that the iteration exceeds the iteration limit number and the algorithm operation ends, otherwise, the iteration continues^26,27^. Because the triangular buffer groove and the U-shaped buffer groove are in an optimal range of depth and plane angle, and each has its own advantages, If the two grooves are combined, it is necessary to optimize the parameters. In this paper, the bird swarm algorithm will be used to optimize the parameters. The algorithm flow chart of the bird swarm optimization algorithm is shown in Fig. 12.

### Parameter optimization of combining buffer tank structure

**Figure 13:**
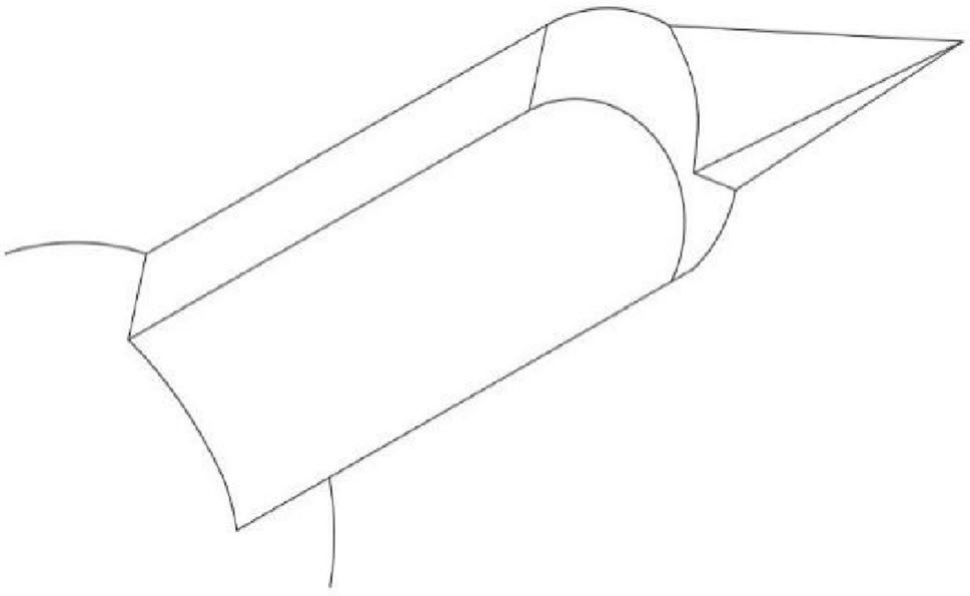
Schematic diagram of combined tank structure.

Because both triangular buffer groove and U-shaped buffer groove have certain defects when working alone, we propose a combined buffer groove with triangular buffer groove at the front end of U-shaped buffer groove. The combined buffer groove is a complex combined structure, which needs to be divided into different stages to establish the mathematical model of the valve port cavity. The structures of two kinds of buffer grooves have been calculated before, and the valve port cavity model of the combined buffer groove is deduced based on the above. As showed in Fig. 13, from the triangular buffer tank stage, the overflow area gradually increases and the pressure impact decreases.

After entering the U-shaped buffer tank, the overflow area changes steadily, and the pressure impact gradually decreases. From the structural aspect, the combined buffer tank has more stable depressurization characteristics than triangular buffer tank and U-shaped buffer tank. The flow area under the combined buffer groove structure can be regarded as the waist groove of the valve sleeve which coincides with the triangular buffer groove and the U buffer groove in turn.

Because the combined buffer groove is a combination of triangular buffer groove and U-shaped buffer groove, its area should be equal to that of both buffer grooves, so the length ratio of the two buffer grooves is 2:3. The area of both should account for 2/5 and 3/5 of the total area respectively. Through MATLAB algorithm calculation, the optimal cutting angle is 72 by comparing triangular buffer groove with U-shaped buffer groove. At this time, the optimal plane angle is between 45 and 63, while the optimal depth h of U-shaped buffer groove should be between 1.5 and 1.7 mm. According to the size proportion of triangular buffer tank and U-shaped buffer tank in the combined buffer tank, the formula of overflow area of the combined buffer tank can be obtained.

According to formulas (15) and (39)

49 $$A = \left\{ \begin{gathered} \frac{1}{2}\left| {\overrightarrow {PQ} \times \overrightarrow {PM} } \right| \hfill \\ \left| {\overrightarrow {KL} } \right| \cdot h \hfill \\ \end{gathered} \right.$$

**Figure 14:**
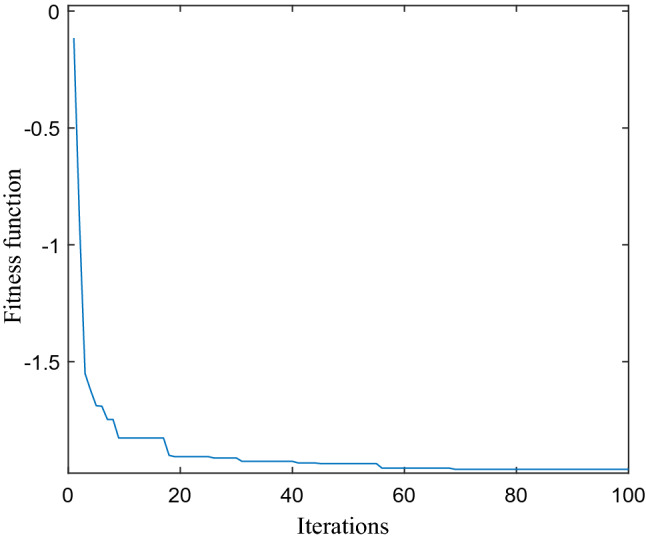
Optimization curve of objective function.

The pressure gradient of the valve port cavity under the combined structure of valve sleeve and valve core is taken as the objective function, the plane angle and depth h are taken as constraint conditions, and the pressure gradient is taken as the objective function. Set the total number to 10, the spatial dimension to 1, the number of iterations to 100, the flight frequency to 8, the cognitive coefficient to 1, the parameters *a*_*1*_ and *a*_*2*_ to 1, and the social evolution coefficient to 1. The parameters of the combined pressure tank are optimized by using the bird swarm optimization algorithm. The objective function is simulated by MATLAB software, and the results are showed in Fig. 14.

**Figure 15:**
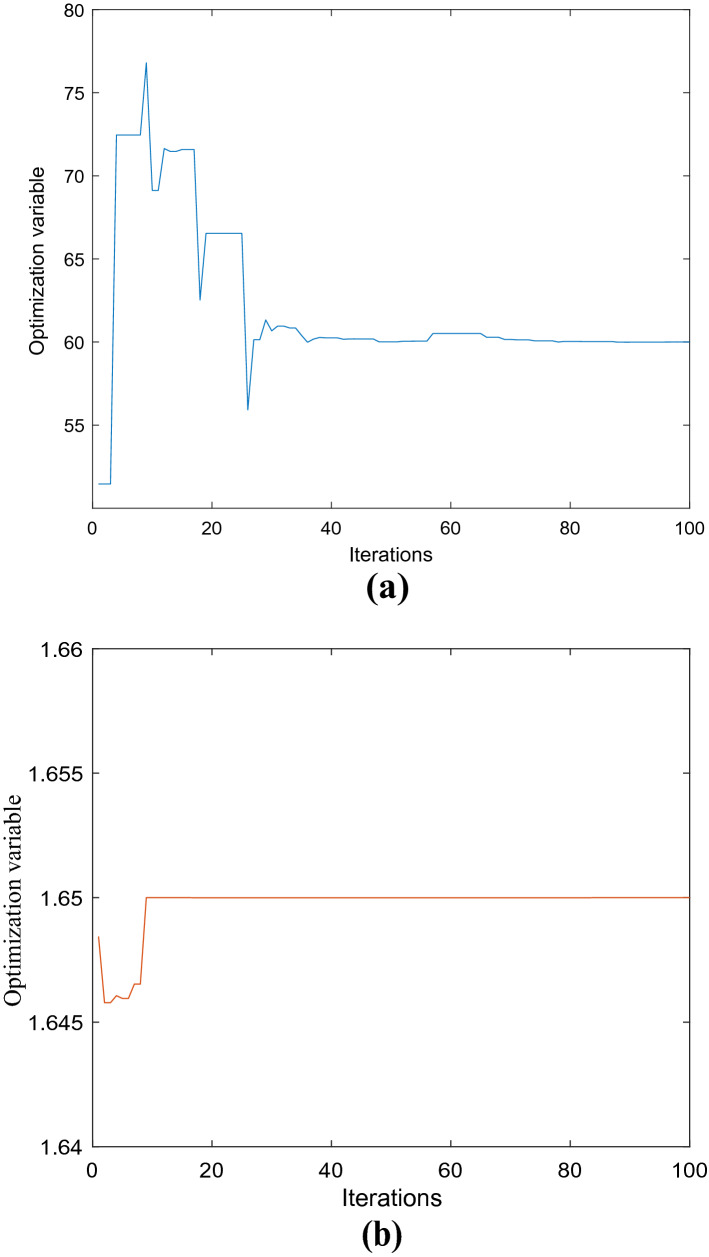
Iterative curve of depth angle and depth optimization.

In order to get the optimal pressure gradient of the valve port volume under the combined structure of valve sleeve and valve core, the introduced fitness function is iterated, and the minimum value of the fitness function is iterated. As shown in Fig. 14, when the iterative calculation reaches the 53rd step, the objective function starts to converge to − 1.94 in the bird swarm algorithm. At this time, the optimization results of plane angle in the range of 45–72 and depth in the range of 1.5–1.7 mm are also obtained. From Fig. 15a, it can be found that when the plane angle is 60, the variable optimization curve tends to be stable in the process of pressure reduction in the valve port cavity under the combined buffer groove structure, and the optimal value of pressure reduction effect is obtained; From Fig. 15b, it is found that when the depth tends to 1.65 mm,the curve is gradually stable, and the best value of the depressurization effect is obtained. It can be seen that when the plane angle of the combined buffer groove is 60 and the depth is 1.65 mm, the pressure reduction effect is the best.

### Fluid–solid coupling simulation of different buffer tank structures

The purpose of optimization is to reduce the stress concentration at the U-shaped notch of the combined structure of the valve sleeve and valve core while ensuring the normal operation of the regulating valve. Compared with the single-objective optimization of triangular buffer slot and U-shaped buffer slot, the multi-objective optimization of the bird swarm algorithm is more likely to obtain a better solution, and the influence of design variables on the objective function is clearer^28,29^. Through the fluid–solid coupling simulation comparison of the combination structures of the valve sleeve and the valve core of non-grooved, triangular buffer groove, U-shaped buffer groove and combined buffer groove, the advantages of the optimal combined buffer groove are analyzed and compared.

### Material parameter setting

**Figure 16:**
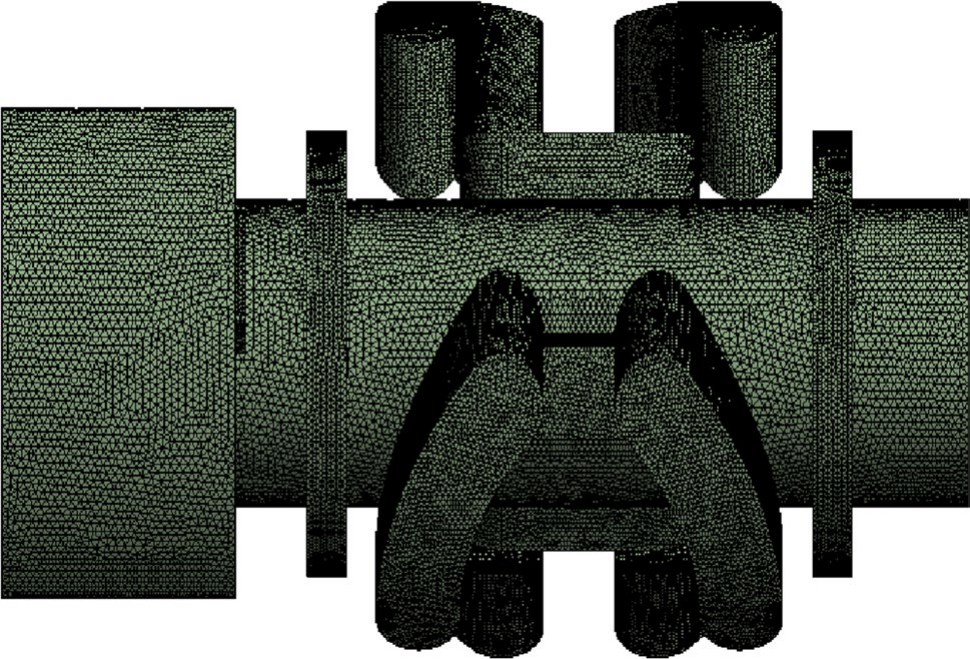
Internal fluid grids of regulating valve.

Under the condition of ensuring the accuracy of numerical calculation, taking into account the complex curved surface structure of the regulating valve, unstructured tetrahedral grids are adopted for the regulating valve.hen, according to the grid independence test, the number of grids generated by fluid in the valve sleeve and valve core combination structure is 1.18 million, which can fully guarantee the accuracy of numerical calculation results and set the expansion layer for the liquid in the valve body, as showed in Fig. 16.

The properties of the fluid material are shown in Table 1, and its interface with the solid is close to the actual model in order to make the subsequent fluid simulation more realistic. In addition, the coupling effect of valve sleeve and valve core combination structure only occurs at the intersection of fluid and solid. Usually, Lagrange motion formula is used to simulate the behavior of solid medium (particle motion), and Euler formula is used to simulate fluid flow (the behavior of fluid in a specific position in space). In the simulation of fluid–solid coupling of regulating valve, considering the fluid–solid coupling effect, any Lagrange Euler formula (ALE) can be used to describe the interaction between fluid and solid medium or fluid and free surface^30,31^. The ALE method is used to deal with the fluid–solid coupling of valve sleeve and valve core combination structure. In “Reference Frame”, the solid is set as Lagrangian and the liquid is set as Eulerian. The geometric model and grid of the valve body and the fluid can overlap, and the related parameters of the regulating valve are shown in Table 2, which can be transmitted to the fluid unit, thus coupling the valve body and the fluid together. Make contact settings for each part of the model, wherein the contact surface between the valve sleeve and the liquid in the valve core is set to be bonded; there is a gap between the valve core and the valve sleeve, and the contact is Frictionless.

**Table 1:** Material characteristics of oil in regulating valve.

Anti-wear hydraulic oil YA-N46		
Density (kg/m^3^)	Dynamic viscosity (Pa s)	Kinematic viscosity (mm^2^/s)
29	0.0245	860

**Table 2:** Material characteristics of valve sleeve and valve core.

Regulating valve parameter			
Density (kg/m^3^)	Modulus of elasticity (N/m^2^)	Poisson's ratio	Specific heat (J/(kg K))
7.88 × 10^3^	2.12 × 10^11^	0.284	460

## Results and analysis

Static structural (ANSYS) is used to analyze the combined structure of the valve core and valve sleeve^32,33^. Through the results of static analysis below, the internal structure of the control valve is further optimized and improved.

**Figure 17:**
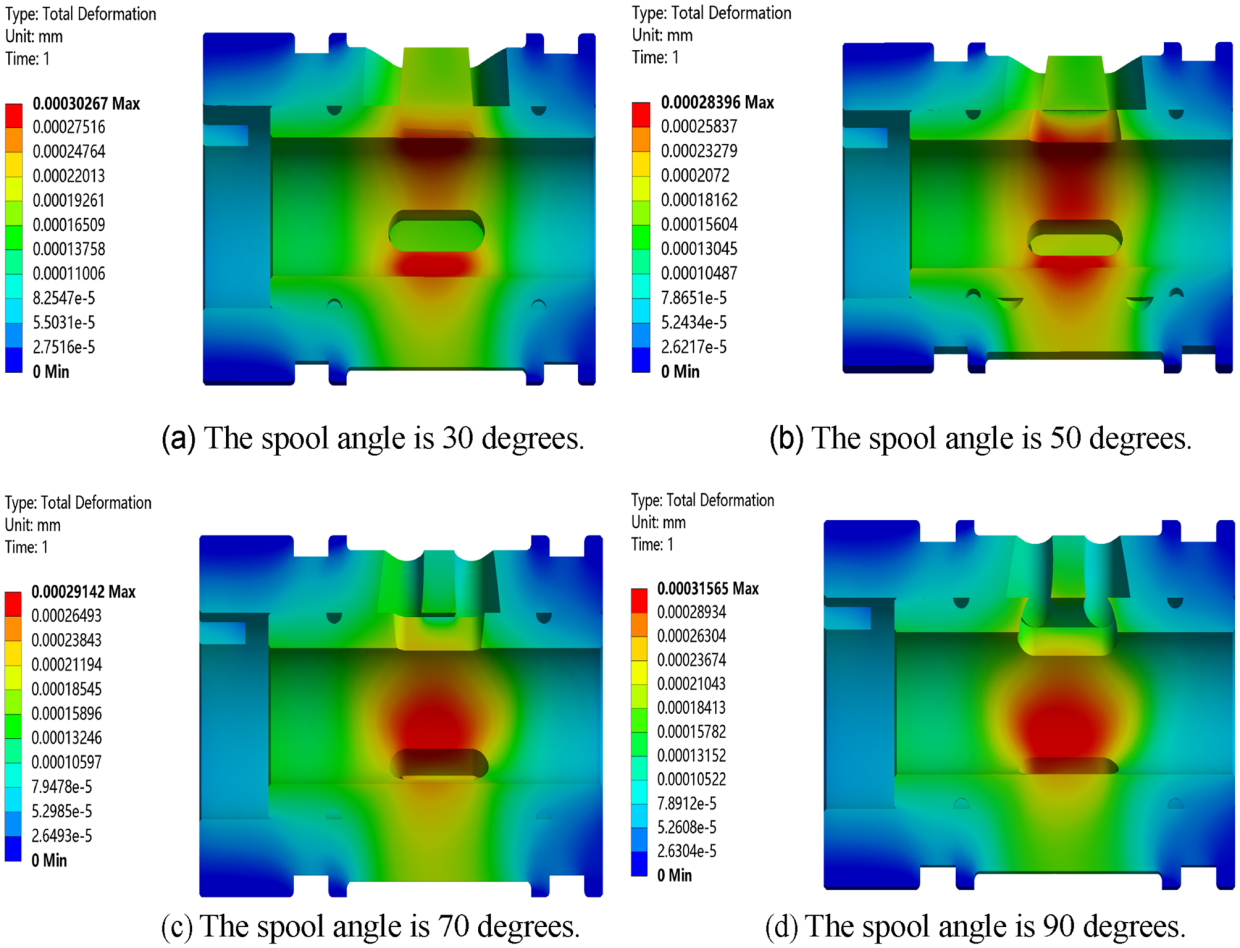
Deformation diagrams of valve sleeve and valve core before implementing buffer groove.

**Figure 18:**
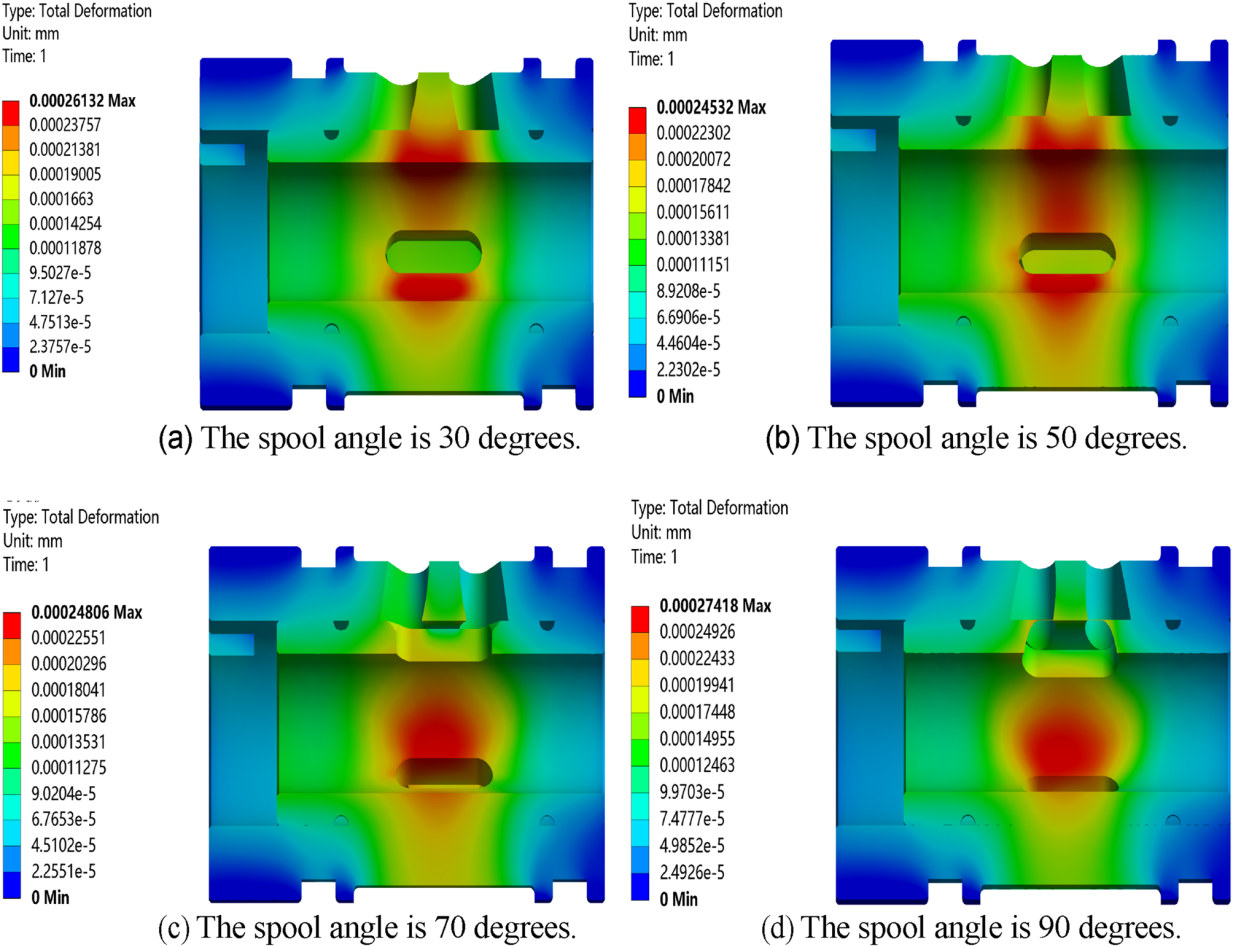
Deformation diagram of valve sleeve and valve core of triangular buffer groove.

**Figure 19:**
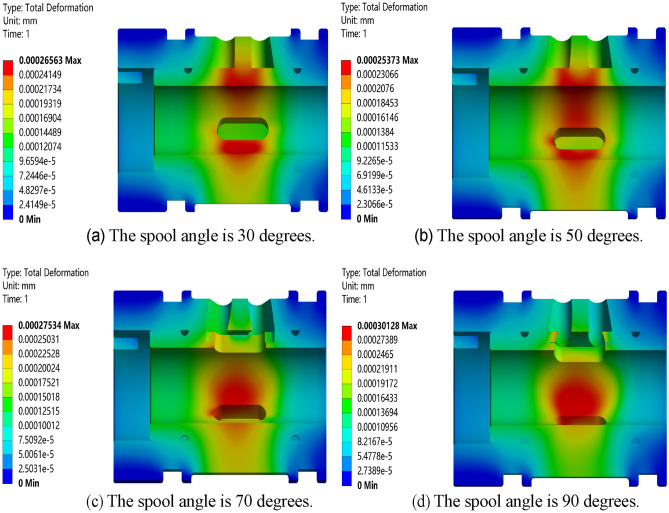
Deformation diagram of valve sleeve and valve core under U-shaped buffer tank.

**Figure 20:**
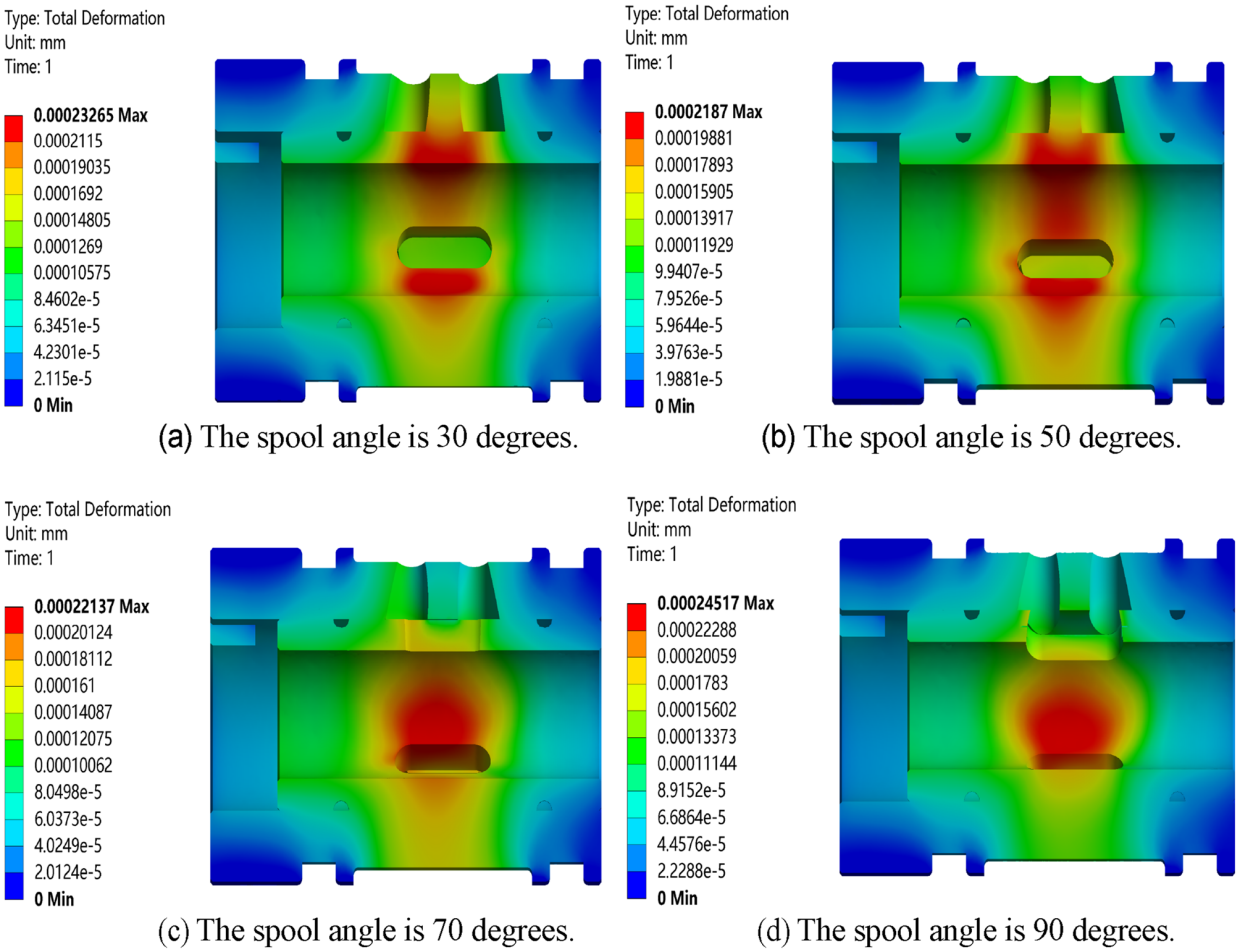
Deformation diagram of valve sleeve and valve core under combined buffer tank.

Carry out static simulation analysis on the valve core and valve sleeve inside the regulating valve, and get the corresponding total deformation cloud map, as showed in Figs. 17, 18, 19 and 20. It can be seen from the figure that the maximum deformation occurs near the valve port of the valve core. With the rotation of the valve core, by comparing the maximum deformation before and after the optimized design, it can be concluded that the combined buffer groove has a remarkable depressurization effect, followed by the triangular buffer groove and the U-shaped buffer groove. From this, it can be concluded that the opening of buffer groove can improve the occurrence of sticking caused by the deformation of the internal valve core and valve sleeve of the regulating valve, and the effect of combining buffer groove is the best.

**Figure 21:**
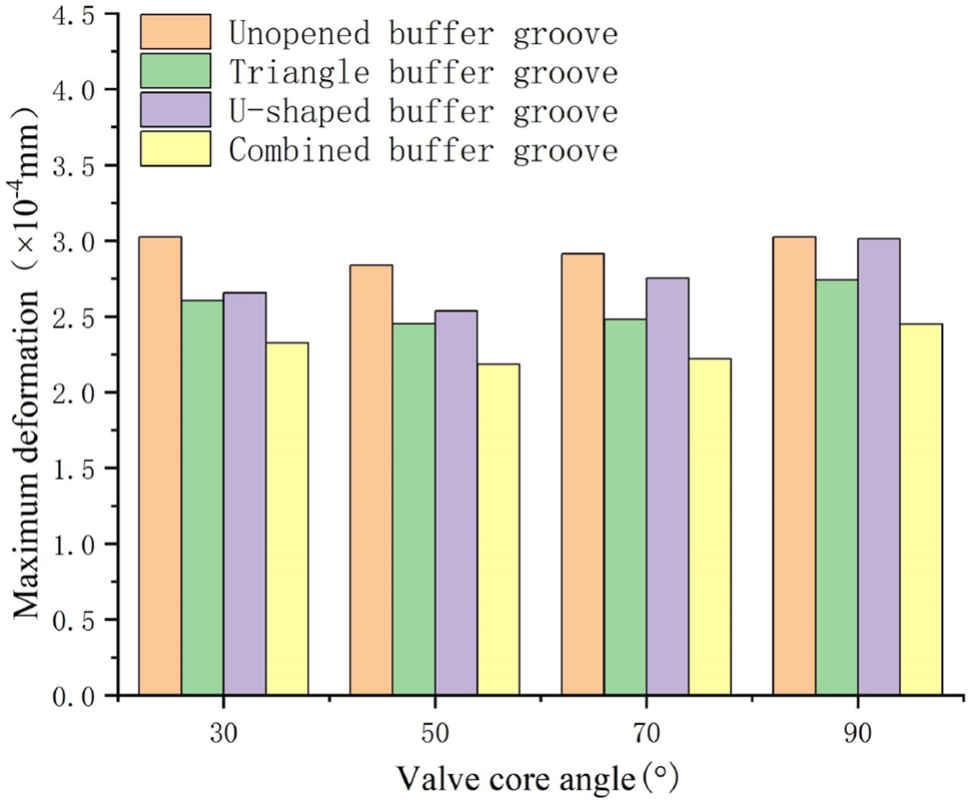
Deformation of valve sleeve and spool in each buffer slot.

Through the deformation diagram obtained by fluid–solid coupling simulation under the structure of non-grooved, triangular buffer groove, U-shaped buffer groove and combined buffer groove, it can be seen that the buffer groove structure on the valve port of the valve core can reduce the stress concentration caused by pressure impact. According to Fig. 21, under the same working condition, the overall deformation of the regulating valve is in the order of combined buffer groove < triangular buffer groove < U-shaped buffer groove < unopened buffer groove, which shows that opening buffer groove can effectively reduce the deformation of the combined structure of valve sleeve and valve core of the regulating valve, and the reduction of combined buffer groove is the most obvious.

## Discussion

Aiming at the phenomenon that the valve core of the micro-flow regulating valve is deformed due to pressure impact, and the valve sleeve is stuck in motion, and to solve the problem that the torque required to drive the valve core to rotate is large, a buffer groove is opened at the valve port of the valve core. In this paper, the structure of buffer tank is optimized, and its parameters are optimized and the improved structure is compared by fluid–solid coupling simulation. The conclusions are as follows.

1. Mathematical models of valve sleeve and valve core of triangular and U-shaped buffer grooves are established respectively, and the pressure gradient curves of them are obtained through MATLAB simulation calculation. Through analysis, it is concluded that if the optimal triangular buffer groove and the optimal structural parameters of U-shaped buffer groove are to be taken simultaneously, the cutting angle should be 72, the plane angle should be in the range of 45–72 and the depth should be 1.5–1.7 mm, and the pressure reduction effect can be obtained at this time.
2. By studying the structure of triangular buffer and U-shaped buffer groove, a combined buffer groove structure is designed to combine the advantages of both. Therefore, taking depth angle and depth as constraints, the pressure gradient of the valve port cavity under the combined buffer groove structure is taken as the objective function. Through the bird swarm optimization algorithm (BSA), it is found that the pressure reduction effect of the combined buffer tank is the best when the plane angle is 60 and the depth is 1.65 mm. At this time, the structural parameters are the optimal values of the depth angle and depth of the combined buffer tank.
3. Through the fluid–solid coupling analysis of the combined structure of valve sleeve and valve core before and after optimization, it is concluded that opening buffer groove can effectively improve the deformation of valve core and compared with U-shaped buffer groove and triangular buffer groove, the combined buffer groove can effectively improve the deformation of the combined structure of valve sleeve and valve core.

Therefore, the excellent structure and parameters of the combined buffer tank are obtained, and the pressure impact of the regulating valve at the key position of the valve port is reduced to the best effect, which provides an effective solution for solving the problem of the locking of the regulating valve when working.

## Data Availability

The datasets used and/or analyzed in this study are available from the corresponding author upon reasonable request.
